# Dietary Inflammatory Index in relation to Type 2 Diabetes: A Meta-Analysis

**DOI:** 10.1155/2022/9953115

**Published:** 2022-02-17

**Authors:** Amir Motamedi, Mohammadreza Askari, Hadis Mozaffari, Reza Homayounfrar, Ali Nikparast, Maryam Lafzi Ghazi, Maryam Mofidi Nejad, Shahab Alizadeh

**Affiliations:** ^1^Student Research Committee, Shiraz University of Medical Sciences, Shiraz, Iran; ^2^Department of Community Nutrition, School of Nutritional Sciences and Dietetics, Tehran University of Medical Sciences, Tehran, Iran; ^3^Faculty of Land and Food Systems, University of British Columbia, Vancouver, Canada; ^4^Noncommunicable Diseases Research Center, Fasa University of Medical Sciences, Fasa, Iran; ^5^National Nutrition and Food Technology Research Institute, Faculty of Nutrition Sciences and Food Technology, Shahid Beheshti University of Medical Sciences, Tehran, Iran; ^6^Department of Exercise Physiology, Central Tehran Branch, Islamic Azad University, Tehran, Iran; ^7^Department of Community Nutrition, School of Nutritional Sciences and Dietetics, Tehran University of Medical Sciences, Tehran, Iran; ^8^Department of Cellular and Molecular Nutrition, School of Nutritional Sciences and Dietetics, Tehran University of Medical Sciences (TUMS), Tehran, Iran

## Abstract

**Background and Aims:**

Epidemiologic studies show a strong association between chronic inflammation and type 2 diabetes (T2D). Diet may also affect the risk of T2D by modulating inflammation. This meta-analysis aimed to assess the relation of dietary inflammatory index (DII) and risk of T2D.

**Methods:**

PubMed and Scopus were systematically searched from their inception to September 2020 to identify relevant studies. Relative risks, hazard ratios, or odds ratios (OR), with their corresponding 95% confidence intervals (95% CI), were calculated and pooled using a random-effects model.

**Results:**

A total of 48 different studies, with a total sample size of 1,687,424 participants, were eligible to be included in this meta-analysis. In the overall analysis, no significant association was observed between DII and risk of T2D (OR = 1.03, 95% CI: 0.91 to 1.15), with significant evidence for heterogeneity (*I*^*2*^ = 96.5%, *P* < 0.001); however, higher DII was identified as being significantly related to increased risk of T2D in high quality studies (OR = 1.58, 95% CI: 1.15 to 2.17). In the stratified analysis by the dietary assessment tool, background disease, and sex of participants, DII showed no significant association with T2D.

**Conclusions:**

Higher DII might be associated with an increased risk of T2D. Additional well-designed studies are required to confirm this finding.

## 1. Introduction

Diabetes is one of the fastest-growing health issues that has reached an alarming level in many societies today [1, 2]. In the last 20 years, the number of adults diagnosed with diabetes almost tripled, from 151 million to over 463 million, and this number is projected to reach 700 million by 2045 [3]. Globally, 4.3 million deaths and USD 760 billion direct health expenditure have been attributed to diabetes in 2019 [3]. Type 2 diabetes (T2D) is contributing to 90–95% of all diabetes cases [3] and is a potential risk factor for cardiovascular disease [4], so it is critical to find a low-cost strategy that can help in its early prevention. The role of the immune system in development of T2D has gained interest in recent years such that a growing number of studies have highlighted the involvement of inflammatory biomarkers in the pathogenesis of T2D [5, 6]. Studies have shown that adiposity, insulin resistance, and hyperglycemia can induce systemic inflammation through stimulating the production of proinflammatory proteins such as C-reactive protein and cytokines including interleukin-1*β* (IL-1*β*), interleukin-6 (IL-6), and tumor necrosis factor-*α* (TNF-*α*) [7]. Additionally, environmental and behavioral factors can augment systemic inflammation in the time of stress [8].

Diet is one of the key modifiable factors which may modulate the systemic inflammation [9]. That is, diet may influence metabolic heath through dietary factors that exhibit anti- or proinflammatory properties [10]. For instance, dietary flavonoid intake has been inversely associated with inflammation [11], whereas saturated fatty acids have shown a positive association [12]. However, studying whole dietary patterns/indices may be better in evaluating the inflammatory effect of diet because such patterns/indices can capture the interaction of multiple nutrients or foods to reflect the complexity of a diet [13]. Dietary diversity index, also known as potential inflammatory of diet, is a literature-derived, population-based dietary index developed by Shivappa et al. [14]. DII includes 45 food parameters (foods, macro- and micronutrients) that are classified into three categories based on their impact on key inflammatory biomarkers: anti-inflammatory, proinflammatory, and inflammatory neutral [14]. Review studies assessing DII in relation to metabolic outcomes have revealed a positive link between DII and obesity [15], metabolic syndrome [16], and cardiovascular diseases [17].

However, to date, there is no meta-analysis assessing diabetes outcomes of DII although two meta-analyses have assessed the association of DII with markers of glucose homeostasis that precede diabetes [18, 19]. These meta-analyses showed that higher DII scores were significantly associated with hyperglycemia, hyperinsulinemia, and insulin resistance measured by HOMA-IR [18, 19]. However, the results were based on a small number of studies (*n* = 3–13) and had high between-study heterogeneity, and the authors omitted important free-text thesaurus terms such as “dietary inflammatory pattern” [18, 19]. Determining the association of DII with T2D may facilitate its use for intervention and provide a basis for assessment of T2D risk at population level. Therefore, given that the association of DII with T2D still remains unclear and controversial in the literature, this meta-analysis aimed to systematically examine and quantify the association of DII with the risk of T2D.

## 2. Methods

The common approach used for this study was reported according to the Preferred Reporting Items for Systematic Reviews and Meta-Analyses (PRISMA) statement [20].

### 2.1. Search Strategy and Selection

A systematic search of peer-reviewed literature in Medline/PubMed was supplemented by hand searching references of eligible studies and relevant reviews. We used broad free-text thesaurus terms and Medical Subject Headings for “dietary inflammatory index” and “type 2 diabetes” to set the search syntax as follows: (((((“dietary inflammatory index” [Title/Abstract]) OR (“inflammatory diet” [Title/Abstract])) OR (“anti-inflammatory diet” [Title/Abstract])) OR (“inflammatory potential of the diet” [Title/Abstract])) OR (“proinflammatory diet” [Title/Abstract])) AND ((((((“diabetes mellitus” [MeSH]) OR (diabetes)) OR (“type 2 diabetes mellitus”)) OR (T2DM)) OR (“non-insulin-dependent diabetes mellitus”)) OR (“diabetes mellitus”)). No limitation was placed on date, and all studies from Medline inception to January 2021 were imported to an EndNote library for screening. Search was conducted by SA.

### 2.2. Inclusion Criteria

This review included eligible observation studies (longitudinal, cross-sectional, and case-control) examining potential inflammatory of diet (exposure) as a function of T2D (outcome) and reported relative risks (RR), hazard ratios (HR), or odds ratios (OR) or provided sufficient data to calculate them; when data were not reported in the studies, we contacted the corresponding author to obtain data. Criteria for exclusion included the following: clinical trials, editorials, books, dissertation, and other grey literature. Data were extracted independently by two reviewers, and disagreements were resolved by discussion.

### 2.3. Screening

All the records were imported to an EndNote library for enhancing the screening process. AM and HM screened titles and abstracts for potential eligibility and removed records based on predefined criteria. In studies with T2D as the secondary outcome, it was difficult to determine eligibility based on title/abstract, so full text of 80% of studies were retrieved for further inspection. Retrieved papers were read in full and all references followed up.

### 2.4. Data Extraction and Quality Assessment

An evidence table with a priori determined headings was used for data extraction of eligible studies as follows: the first author's name, geographical setting and population, year of publication, sex and mean age of participants, sample size, DII scoring system(s) and assessment tool(s), T2D assessment tool, DII comparison cut-points, documented effect sizes, and covariates adjusted, if available. Data were extracted independently by two reviewers, and in case of disagreement, a third reviewer was involved.

Two reviewers (SA and HM) separately filled out Newcastle–Ottawa Scale (NOS), one of the best tools for quality appraisal of observational studies, for included studies. Cross-sectional and case-control studies were assigned a maximum of 10 asterisks for the three domains of selection (0–4), comparability (0–2), and assessment of exposure (0–3). Each cohort study was also given a maximum of 10 asterisks for parameters in the three domains of selection (0–5), comparability (0–2), and assessment of outcome (0–3). Final quality scores were determined based on the number of criteria received “*∗*”: 7–9 (high quality); 4–6 (medium quality); 1–3 (low quality).

### 2.5. Data Synthesis and Analysis

Studies reporting the association of DII with T2D using odds ratios, hazard ratios, or relative risks were considered as eligible for qualitative synthesis. However, for quantitative synthesis, log-transformed risk ratios as well as corresponding standard errors (SEs) were obtained from ORs, RRs, or HRs extracted from models that were adjusted for the highest number of covariates. If the study did not report these three risk ratios directly, we used the raw data (total sample size and cases) provided by the study to estimate the unadjusted ORs. Due to great variety in the included studies, particularly in terms of DII scoring system, a random-effects model was used for pooling risk estimates on a forest plot regardless of the presence or absence of heterogeneity (*I*^*2*^ values). *I*^*2*^ > 50% together with *P* < 0.10 was used to determine statistically significant heterogeneity. Subgroup analyses using random-effects models investigated potential sources of variation that were due to reasons other than chance. Potential sources examined were quality of studies, dietary assessment tool, and sex of participants. The possibility of publication bias was assessed by conducting Egger's test (*P* value set at 0.05) and constructing funnel plots for visual inspection. Metaregression analysis was conducted to assess if pooled estimates were affected by age of participants. Sensitivity analysis was conducted to assess the robustness of the synthesized results. All analyses were done using Stata, version 13 (StataCorp, College Station, TX). Pooled estimates' significance was set at *P* < 0.05.

## 3. Results

**Figure 1:**
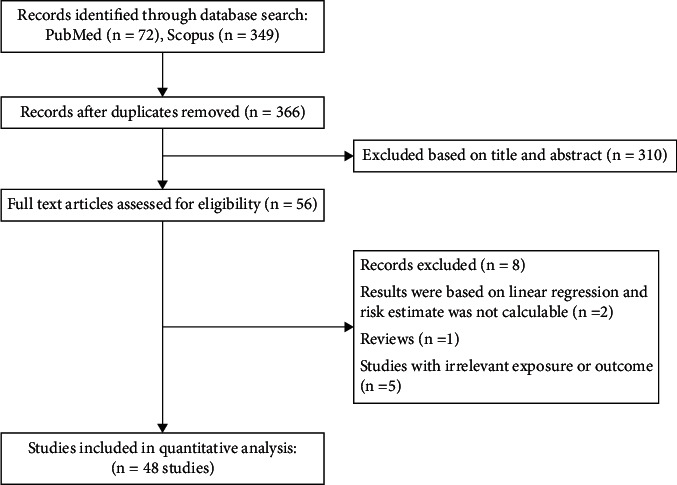
Flow chart of study.

The preliminary search through databases yielded a total of 421 publications. After excluding duplicate (*n* = 55) and irrelevant publications (*n* = 310) based on title and abstract, 56 articles underwent full-text screening. Among them, 8 further studies were excluded since they were reviews, had irrelevant exposure or outcome, or reported results according to the linear regression analysis (beta coefficient), and thus their risk estimates were not calculable [21, 22]. Eventually, the present meta-analysis included a total of 48 different studies with 55 data sets [23–70] including a total sample size of 1,687,424 participants. For some studies, different effect sizes had been reported in their stratified analyses; we extracted all effect sizes for such studies. The flow chart of the study showing the process of screening is presented in Figure 1. Studies were published during 2013 to 2020, and the sample size of the included publications varied between 22 and 533256 subjects. Among the included studies, there were 8 high quality studies reporting the adjusted risk estimate for T2D associated with DII as the primary outcome [32, 33, 39, 50–52, 59, 63], while for other studies [23–31, 34–38, 40–49, 53–58, 60–62, 64–70], T2D was a secondary outcome and the crude odds ratio for T2D was calculated based on the frequency of subjects with T2D in the highest category of DII, compared with subjects in the lowest category. DII was calculated with the use of 7-day dietary record in 3 studies [31, 44, 50], 24-hour dietary recall in 11 studies [35, 37, 46, 51, 54, 55, 62–64, 68, 70], dietary history questionnaire in 2 studies [45, 52], and food frequency questionnaire (FFQ) in the remaining studies. Moreover, data for men was reported in 7 studies [28, 36, 44, 50, 53, 54, 64] and for women was reported in 10 studies [24, 28, 34, 36, 38, 49, 52, 54, 64, 67], and other studies reported results for a combination of both sexes. The quality of the included publications was considered as high in 8 studies [32, 33, 39, 50–52, 59, 63] and low in 40 studies [23–31, 34–38, 40–49, 53–58, 60–62, 64–70].  Table 1 represents other characteristics of the analyzed studies.

### 3.1. Quantitative Synthesis of Data

**Figure 2:**
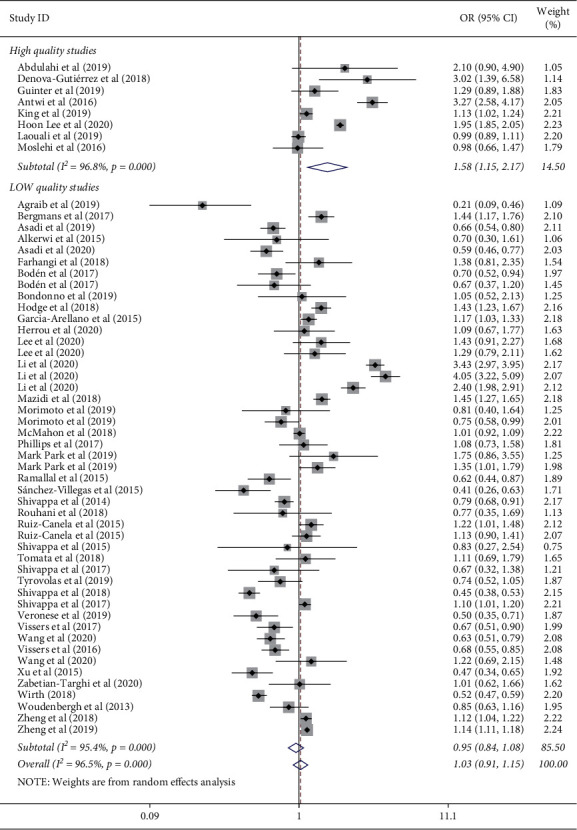
Meta-analysis for the association of dietary inflammatory index and risk of type 2 diabetes stratified by the quality of studies.

The results for the overall and subgroup analysis are shown in Table 2. In the overall analysis, when all eligible publications were pooled, no significant association was found between DII and risk of T2D (OR = 1.03, 95% CI: 0.91 to 1.15), with significant evidence for heterogeneity (*I*^*2*^ = 96.5%, *P* < 0.001); however, higher DII was identified as being significantly related to a 58% increased risk of T2D in high quality studies (OR = 1.58, 95% CI: 1.15 to 2.17) (Figure 2). No significant relationship was observed between DII and risk of T2D in the subgroup analysis based on the dietary assessment tool and sex of participants (Table 2).

### 3.2. Metaregression and Publication Bias

**Figure 3:**
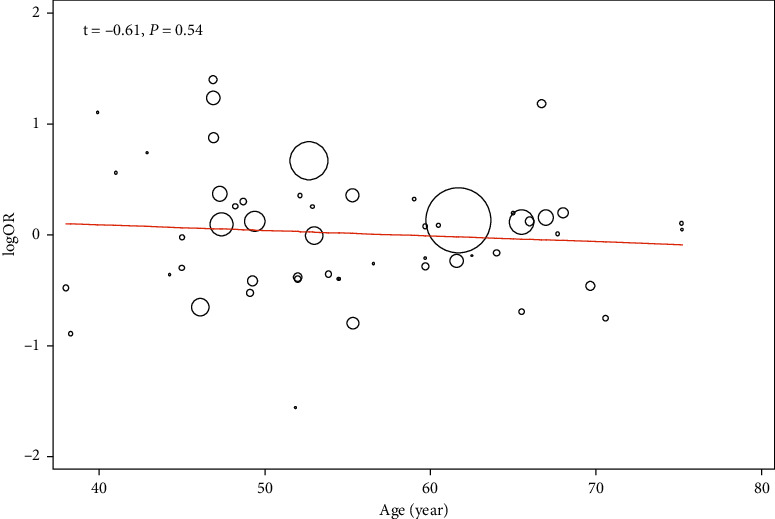
Metaregression analysis for the effect of age on the relation of dietary inflammatory index to risk of type 2 diabetes.

**Figure 4:**
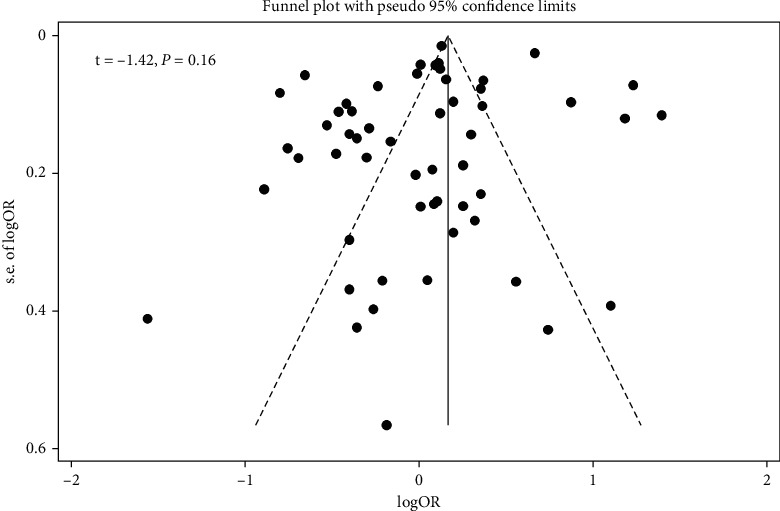
Funnel plot for publication bias.

In the metaregression analysis, age of participants did not affect the relation of DII to T2D (*t* = 0.62, *P* = 0.54) (Figure 3). Furthermore, no evidence of publication bias was found based on the Egger test (*t* = - 1.42, *P* = 0.16) (Figure 4).

### 3.3. Sensitivity Analysis

**Figure 5:**
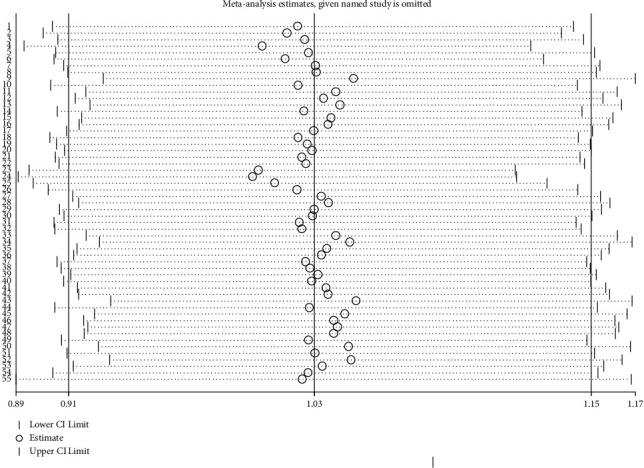
Sensitivity analysis by omitting one study at a time and reanalyzing other studies.

Sensitivity analysis by omitting one study at a time did not significantly change the results, showing the reliability of the findings (Figure 5).

## 4. Discussion

Studies exploring the relation of DII to the risk of T2M have yielded inconclusive findings. This meta-analysis aimed to resolve these inconsistencies by pooling the available observational studies. The pooled effect size of high quality studies showed that higher DII is significantly related to the increased risk of T2D, although this finding was not supported in the stratified analyses by dietary assessment tool and sex of participants.

Long time exposure to low-grade inflammation could result in an elevation in the risk of obesity and its-related metabolic complications such as T2D [71]. The relation of systemic inflammatory markers to T2D has been well established. Diet is also suggested as a main contributor to the balance that affects the overall inflammatory response at various sites in chronic conditions. Investigations have identified a positive association between unhealthy Western dietary patterns—featured mainly by lower consumption of fish, fruits, vegetables, fiber, and whole grains and higher intake of red and processed meat, sugar, solid fat, and fast foods—and inflammation [72] as well as T2M [73, 74]. Moreover, multiple prospective cohort studies have shown a negative relationship between the adherence to Mediterranean diet (with anti-inflammatory effects) [75] and the risk of T2M [76]. In this meta-analysis, we found a direct relationship between inflammatory potential of a diet, assessed by DII, and risk of T2D. DII is closely correlated to circulating inflammatory biomarkers [77], a potential pathway involved in the T2D pathogenesis [78]. The result in our analysis is supported by previous studies examining the association between DII and T2M; in the study by Denova-Gutiérrez et al. [39] on 1174 Mexican adults, after controlling potential confounding factors including body mass index (BMI), it was revealed that people with the highest score for the DII had a 3-fold increased risk of T2D, compared with individuals with the lowest scores of DII. Similarly, a recent study [79] found that individuals adhering to a proinflammatory diet had a 18-fold higher odds of prediabetes, compared to those consuming an anti-inflammatory diet. In the cross-sectional analysis of 4434 adults with age ≥20 years in the National Health and Nutrition Examination Survey [51], the mean DII scores in people with T2D and with hemoglobin A1c (HbA1c) > 6.5% were significantly higher than those without T2D and those with HbA1c <6.5%, proposing that DII is a significant predictor of diabetes and its severity, so that with 1 point increase in the DII score, odds of having diabetes and HbA1c higher than 9% increased by 13% and 43%, respectively. Moreover, HbA1c was significantly related to an increased CRP [51]. More recent studies have yielded additional support for our finding; DII is reported to be directly related to all markers of T2D risk, including fasting insulin, fasting glucose HbA1c, homeostasis model assessment index for insulin resistance (HOMA2-IR), and two-hour glucose levels [21]. The anti-inflammatory dietary pattern was also negatively associated with CRP and lower odds of T2D in a recent study in the National Diet and Nutrition Survey on a total of 1531 British adults [80]. Such findings support the results of the present meta-analysis that diet-induced inflammation increased the odds of T2D. Nevertheless, the cross-sectional study from the Tehran Lipid and Glucose Study including 2975 Iranian adults [33] found no significant relationship between DII and impaired fasting glucose, insulin resistance, and T2D, while DII had a positive weak relation only to 2-hour plasma glucose (2h-PG). These discrepancies might be derived from differences in study design, background disease, types and number of dietary components applied to compute the DII, level of adjustments for covariates, and most importantly quality of studies. It should be considered that the null association of DII with T2D in the overall and subgroup analysis is due to the high number of low quality studies in the overall and subgroup analysis, in which T2D was not a primary outcome.

The precise mechanism by which diet-related inflammation might elevate the odds of T2D is not well known although it is well established that T2D is fostered in a proinflammatory setting [71]. A proinflammatory diet might contribute to the risk of T2D by elevating circulating levels of inflammatory cytokines (e.g., interferon *γ*, IL-1, IL-6, IL-8, CRP, and TNF-*α*), which can lead to insulin resistance [6, 77]. It has been suggested that some nutrients and foods could have immunomodulatory impacts and reduce inflammation, thereby improving insulin resistance and beta cell function [81]. In this sense, most of the frequently used food items in the proinflammatory diet (trans-fatty acids, saturated fatty acids, red and processed meat, and energy intake) have been linked to inflammatory markers [82–84] and T2M [85, 86]. However, low consumption of n-3 fatty acids, vegetables, dietary fiber, and fruits in a diet with low DII score has been linked to elevated risk of T2D [87, 88], possibly mediated by an increase in inflammatory markers [89]. In addition, some studies [21, 52], but not all [39], have revealed that the relation of DII to T2D is partly mediated by BMI so that a diet with high inflammatory potential could increase BMI, which is one of the strongest risk factors for T2D. Furthermore, an anti-inflammatory diet has been shown to be inversely related to glycemic index (GI) score [90]; on the other hand, a diet with low GI leads to weight loss, along with a reduction [91] in proinflammatory mediators [92] and improvement in insulin sensitivity [93], justifying the link of high DII diets to T2D.

The present study is the first meta-analysis to comprehensively investigate the relation of DII to T2D. The results have significant clinical implications as targeting the DII components could be a useful strategy to decrease the risk of T2D. As a strength point, there was no significant evidence for publication bias. However, some limitations of our meta-analysis should be considered. First, T2D was a secondary outcome in the majority of the included studies, and we calculated crude effect size for such studies, which may increase their susceptibility to bias. Nevertheless, to resolve this issue, we performed subgroup analysis based on the study quality to obtain a more reliable conclusion. Second, a significant heterogeneity was detected across the publications; despite that, we applied random-effects analysis to consider the observed heterogeneity. This heterogeneity can be derived from differences in genetic background, background disease, level of adjustment for covariates, sample size, and quality of studies. Subgroup analysis found that dietary questionnaire used to calculate DII was a potential source of heterogeneity, showing that differences in dietary assessment tool among previous publications may partly justify the detected heterogeneity. Third, the analyzed publications were observational in design, which are subject to selection bias, and causality could not be inferred. Finally, number and type of food components applied for the computation of the DII varied across studies, and standardizing the intake of each food components according to world mean and standard deviation (SD) complicates the comparability of DII across different populations, which may be a source of inconsistent findings in the prior studies.

In conclusion, this meta-analysis indicated that adherence to a proinflammatory diet may increase the risk of T2D. Thus, the recommendation of a healthy dietary pattern may decrease diet-induced inflammation and subsequently lower the risk of T2D. Additional studies are required to identify whether a diet that particularly targets the DII components could be useful clinically to decrease the development of T2D.

## Figures and Tables

**Table 1:** Characteristics of studies.

Population									
Reference	Year	Study design	Location	No. of participants	Sex	Age (range or mean ± SD)	Exposure assessment	Outcome assessment	Diabetes was the primary outcome, and analyses were adjusted for potential confounders
Woudenbergh et al.	2013	Cross-sectional	Netherlands	1024	M/F	64 ± 9	FFQ	FPG-2-h, -HbA1c	No
Shivappa et al.	2014	Cross-sectional	USA	34703	F	61.58 ± 4.2	FFQ	Self-report	No
Alkerwi et al.	2015	Cross-sectional	Luxembourg	1352	M/F	44.25 ± 0.8	FFQ	Medication use	No
Garcia-Arellano et al.	2015	Cross-sectional	Spain	7216	M/F	67 ± 6	FFQ	Medical record	No
Ramallal et al.	2015	Cross-sectional	Spain	18794	M/F	38 ± 12	FFQ	Medical record	No
Sánchez-Villegas et al.	2015	Cross-sectional	Spain	15093	M/F	38.28	FFQ	Medical record	No
Ruiz-Canela et al.	2015	Cross-sectional	Spain	4145	F	68 ± 6	FFQ	Medical record	No
Ruiz-Canela et al.	2015	Cross-sectional	Spain	3091	M	66 ± 7	FFQ	Medical record	No
Shivappa et al.	2015	Case-control	Italy	978	M/F	62.5	FFQ	Medical record	No
Xu et al.	2015	Cross-sectional	Sweden	1942	M/F	70.6 ± 0.6	7-day dietary record	FPG ≥ 126 mg/dL, 2 h glucose ≥ 200 mg/dL, hypoglycemic agents or insulin use	No
Antwi et al.	2016	Case-control	USA	2573	M/F	66.7 ± 10.3	FFQ	Medical records	Yes
Moslehi et al.	2016	Cross-sectional	Iran	2975	M/F	45 ± 11.7	FFQ	FPG ≥ 126 mg/dL, 2 h glucose ≥ 200 mg/dL, use of antidiabetic medication	Yes
Vissers et al.	2016	Cross-sectional	Australia	6972	F	52 ± 1	FFQ	Doctor-diagnosed	No
Bergmans et al.	2017	Cross-sectional	USA	11592	M/F	20-80	24-hour diet recall	Self-reported history	No
Bodén et al.	2017	Case-control	Sweden	5284	M	53.85 ± 7.36	FFQ	Self-report+FPG ≥ 7.0 mmol/L or 2 h glucose ≥ 11.1 mmol/L	No
Bodén et al.	2017	Case-control	Sweden	1660	F	54.47 ± 8.28	FFQ	Self-report+FPG ≥ 7.0 mmol/L or 2 h glucose ≥ 11.1 mmol/L	No
Phillips et al.	2017	Cross-sectional	Ireland	2047	M/F	59.7 ± 0.17	FFQ	FPG ≥ 7 mmol/L or doctor-diagnosed diabetes	No
Shivappa et al. 1	2017	Cross-sectional	Germany	1297	M	54.5 ± 5.8	7-day dietary record	Self-report	No
Shivappa et al. 2	2017	Cross-sectional	USA	12366	M/F	47.4 ± 19.1	24-hour diet recall	Self-report	No
Vissers et al.	2017	Cross-sectional	Australia	7169	F	52 ± 1	FFQ	Doctor-diagnosed	No
Denova-Gutiérrez et al.	2018	Cross-sectional	Mexico	1174	M/F	39.9 ± 0.48	FFQ	FPG ≥ 126 mg/dL and HbA1c> 6.5%	Yes
Farhangi et al.	2018	Cross-sectional	Iran	454	M/F	59.02 ± 9.07	FFQ	-	No
Hodge et al.	2018	Cross-sectional	Australia	39532	M/F	55.3 ± 8.5	FFQ	Structured interview	No
Mazidi et al.	2018	Cross-sectional	USA	21 649	M/F	47.3 ± 0.25	24-hour diet recall	Questionnaire	No
McMahon et al.	2018	Cross-sectional	USA	40161	M	45-69	FFQ	Questionnaire and medical record	No
Rouhani et al.	2018	Cross-sectional	Iran	221	M/F	56.57 ± 15.32	FFQ	Self-report	No
Tomata et al.	2018	Cross-sectional	Japan	793	M/F	75.2 ± 4.5	Dietary history questionnaire	Self-report	No
Shivappa et al.	2018	Cross-sectional	Italy	20823	M/F	55.32 ± 11.6	FFQ	FPG ≥ 126 mg/dL, medication use	No
Wirth	2018	Cross-sectional	USA	26046	M/F	46.1 ± 0.29	24-hour diet recall	Self-report	No
Zheng et al.	2018	Cross-sectional	USA	101449	M/F	65.52 ± 0.04	FFQ	Medical history	No
Abdulahi et al.	2019	Cross-sectional	Iran	300	M/F	42.9 ± 10.9	FFQ	FPG ≥ 126 mg/dL	Yes
Guinter et al.	2019	Cohort	USA	6016	M	48.2 ± 10.02	3-day diet record	Self-report of medication use, self-reports by their personal physician	Yes
King et al.	2019	Cross-sectional	USA	4434	M/F	49.4	24-hour diet recall	HbA1c (%)> 6.5, self-report	Yes
Laouali et al.	2019	Cohort	France	70991	F	53 ± 6.7	Dietary history questionnaire	FPG ≥ 7.0 mmol/L or random glucose ≥11.1 mmol/L at diagnosis, use of a glucose-lowering medication, or HbA1c level ≥53 mmol/mol (7.0%)	Yes
Agraib et al.	2019	Case-control	Jordan	388	M/F	51.85 ± 10.2	FFQ	Interview-based standardized questionnaire	No
Asadi et al.	2019	Cross-sectional	Iran	4672	M/F	49.25 ± 7.85	FFQ	-	No
Bondonno et al.	2019	Cross-sectional	Australia	1422	F	75.2 ± 2.7	FFQ	Medical history	No
Morimoto et al.	2019	Cross-sectional	Brazil	684	M	59.7 ± 13.5	24-hour diet recall	Medical history	No
Morimoto et al.	2019	Cross-sectional	Brazil	1585	F	59.7 ± 13.5	24-hour diet recall	Medical history	No
Mark Park et al.	2019	Cross-sectional	USA	1815	M/F	41	24-hour diet recall	FPG ≥ 100 mg/dL or antidiabetic medication use, HOMA-IR ≥ 90th	No
Mark Park et al.	2019	Cross-sectional	USA	1918	M/F	48.7	24-hour diet recall	FPG ≥ 100 mg/dL or antidiabetic medication use, HOMA-IR ≥ 90th	No
Tyrovolas et al.	2019	Cross-sectional	Greece	3042	M/F	45 ± 14	FFQ	Self-report	No
Veronese et al.	2019	Cross-sectional	Italy	1565	M/F	65.5 ± 8.9	FFQ	Self-report	No
Zheng et al.	2019	Cross-sectional	USA	533256	M/F	61.68 ± 0.02	FFQ	Self-report	No
Hoon Lee et al.	2020	Cross-sectional	USA	19666	M/F	52.7 ± 9.6	FFQ	Self-report	Yes
Asadi et al.	2020	Cross-sectional	Iran	4365	M/F	49.14 ± 7.88	FFQ	-	No
Herrou et al.	2020	Cross-sectional	France	15096	M/F	60.5 ± 5.88	24-hour diet recall	Medical record	No
Lee et al.	2020	Cross-sectional	Korea	1712	M	52.14 ± 0.2	24-hour diet recall	-	No
Lee et al.	2020	Cross-sectional	Korea	2473	F	52.87 ± 0.2	24-hour diet recall	-	No
Li et al.	2020	Cross-sectional	USA	210145	M/F	46.9 ± 9.2	FFQ	Self-report	No
Li et al.	2020	Cross-sectional	USA	210145	M/F	46.9 ± 9.2	FFQ	Self-report	No
Li et al.	2020	Cross-sectional	USA	210145	M/F	46.9 ± 9.2	FFQ	Self-report	No
Wang et al.	2020	Cross-sectional	USA	6893	M/F	69.66 ± 0.3	24-hour diet recall	FPG ≥ 126 mg/dL, 2 h glucose ≥200 mg/dL, self-report, insulin use	No
Wang et al.	2020	Cross-sectional	China	1064	F	65 ± 0.5	FFQ	Medical history	No
Zabetian-Targhi et al.	2020	Cross-sectional	Australia	706	M/F	67.7 ± 6.9	FFQ	RPG ≥ 199.8 mg/dL, FPG ≥ 126 mg/dL, 2 h glucose ≥199.8 mg/dL	No

M: male; F: female; FFQ: food frequency questionnaire; FPG: fasting plasma glucose; RPG: random plasma glucose; HbA1c: hemoglobin A1c; HOMA-IR: homeostatic model assessment for insulin resistance

**Table 2:** Subgroup analysis for the association between dietary inflammatory index and type 2 diabetes.

Subgroup by	Effect sizes^*∗*^ (*n*)	*I* ^*2*^ (%)	*P* heterogeneity	OR (95% CI)
All studies	55	96.5	≤0.001	1.03 (0.91–1.15)
Study quality				
High quality	8	96.8	≤0.001	1.58 (1.15–2.17)
Low quality	47	96.4	≤0.001	0.95 (0.84–1.08)
Sex				
Male	7	51.3	0.05	1.01 (0.85–1.19)
Female	10	72.1	≤0.001	0.88 (0.75–1.03)
Both	38	97.3	≤0.001	1.07 (0.92–1.24)
Dietary assessment				
24-hour recall	13	94.4	≤0.001	1.05 (0.84–1.31)
7-day dietary record	3	87.5	≤0.001	0.74 (0.36–1.52)
Food frequency questionnaire	37	97.0	≤0.001	1.04 (0.89–1.21)
Dilatory history questionnaire	2	0.0	0.064	1.00 (0.89–1.11)

^*∗*^There was more than 1 effect size for some studies.

## Data Availability

All data and codes are available by contacting the corresponding author.
